# Study protocol: Asking QUestions about Alcohol in pregnancy (AQUA): a longitudinal cohort study of fetal effects of low to moderate alcohol exposure

**DOI:** 10.1186/1471-2393-14-302

**Published:** 2014-09-03

**Authors:** Evelyne Muggli, Colleen O’Leary, Della Forster, Peter Anderson, Sharon Lewis, Cate Nagle, Jeffrey M Craig, Susan Donath, Elizabeth Elliott, Jane Halliday

**Affiliations:** Murdoch Childrens Research Institute, The Royal Children’s Hospital, Parkville, 3052 Victoria Australia; Department of Paediatrics, The University of Melbourne, Parkville, 3052 Victoria Australia; Telethon Kids Institute, Perth, 6845 Western Australia Australia; Faculty of Health Sciences, School of Nursing and Midwifery, Judith Lumley Centre, La Trobe University, Melbourne, 3000 Victoria Australia; The Royal Women’s Hospital, Parkville, 3052 Victoria Australia; School of Nursing and Midwifery, Quality and Patient Safety Strategic Research Centre, Deakin University, Geelong, 3220 Victoria Australia; Women’s and Children’s Division, Western Health, St Albans, 3021 Victoria Australia; Paediatrics & Child Health, Children’s Hospital Westmead, The University of Sydney, Sydney, 2006 New South Wales Australia

**Keywords:** Prenatal alcohol exposure, Fetal alcohol spectrum disorders, Cohort studies, Epidemiology, Pregnancy, Child health, Genetics, Epigenetics

## Abstract

**Background:**

Despite extensive research, a direct correlation between low to moderate prenatal alcohol exposure (PAE) and Fetal Alcohol Spectrum Disorders has been elusive. Conflicting results are attributed to a lack of accurate and detailed data on PAE and incomplete information on contributing factors. The public health effectiveness of policies recommending complete abstinence from alcohol during pregnancy is challenged by the high frequency of unplanned pregnancies, where many women consumed some alcohol prior to pregnancy recognition. There is a need for research evidence emphasizing timing and dosage of PAE and its effects on child development.

**Methods/Design:**

Asking QUestions about Alcohol (AQUA) is a longitudinal cohort aiming to clarify the complex effects of low to moderate PAE using specifically developed and tested questions incorporating dose, pattern and timing of exposure. From 2011, 2146 pregnant women completed a questionnaire at 8-18 weeks of pregnancy. Further prenatal data collection took place via a questionnaire at 26-28 weeks and 35 weeks gestation. Extensive information was obtained on a large number of risk factors to assist in understanding the heterogeneous nature of PAE effects. 1571 women (73%) completed all three pregnancy questionnaires. A biobank of DNA from maternal and infant buccal cells, placental biopsies and cord blood mononuclear cells will be used to examine epigenetic state at birth as well as genetic factors in the mother and child. Participants will be followed up at 12 and 24 months after birth to assess child health and measure infant behavioural and sensory difficulties, as well as family environment and parenting styles. A subgroup of the cohort will have 3D facial photography of their child at 12 months and a comprehensive developmental assessment (Bayley Scales of Infant & Toddler Development, Bayley-III) at two years of age.

**Discussion:**

Using detailed, prospective methods of data collection, the AQUA study will comprehensively examine the effects of low to moderate alcohol consumption throughout pregnancy on child health and development, including the role of key mediators and confounders. These data will ultimately contribute to policy review and development, health professional education and information about alcohol consumption for pregnant women in the future.

## Background

It is well recognised that heavy and chronic alcohol consumption in pregnancy is associated with Fetal Alcohol Spectrum Disorder (FASD), a major preventable cause of health and developmental problems in children. FASD encompasses generalised neurodevelopmental impairments including lower IQ, attention difficulties, memory problems, slow processing speed, executive dysfunction, and emotional-behavioural problems [1, 2]. There are, however, conflicting reports of the effect of low to moderate doses of prenatal alcohol exposure (PAE) and any putative association is complex as it is difficult to separate aetiological effects of PAE from other variables that influence childhood behaviour [3–10]. Further, the heterogeneity in findings has in part been attributed to the difficulty in capturing and categorising low to moderate PAE accurately [3, 11–13].

### Public health significance

The lack of clarity on the effects of low to moderate PAE has resulted in policies and guidelines that recommend complete abstinence from alcohol during pregnancy as the safest option [14, 15]. The public health effectiveness of this approach is limited by the high frequency of unplanned pregnancies, with many women having consumed alcohol around the time of conception and even well into the pregnancy before knowing they were pregnant [16–18]. Therefore, there is a need for evidence to better inform pregnant women about the impact of PAE in this critical stage of embryonic development, particularly for low to moderate exposures.

### Objectives

1. To survey mothers at each trimester of pregnancy using a new approach to measure dose and timing of low to moderate alcohol consumption, while collecting important co-factors that are likely to influence outcomes associated with PAE.
2. To survey mothers at 12 and 24 months postpartum to measure parental report of offspring health and development.
3. To collect biosamples at birth for the study of genetic and epigenetic factors in relation to measurements of offspring health and development.
4. To conduct clinical assessments of children at 12 months (facial morphology) and at 24 months (neurodevelopment) in a representative sub-sample of participants from each PAE group.

### Hypothesis

Facial dysmorphology (measured at 12 months of age) and neurodevelopmental delay (measured at 24 months) represent a continuum of subtle fetal alcohol effects, and will be evident in young children exposed to low and moderate doses of alcohol in the first trimester of pregnancy. Such effects will be exacerbated or ameliorated by explanatory factors, including epigenetics.

## Methods

### Study design and study population

AQUA (Asking QUestions about Alcohol) is a longitudinal cohort study designed by Australian researchers who are expert in the field of pregnancy alcohol research, clinical care, epidemiology, genetics, epigenetics, craniofacial analysis, neurodevelopment and FASD.

All women making their first appointment for antenatal care at one of seven metropolitan public hospitals between 25 July 2011 and 30 July 2012 were eligible to participate in the study if they were less than 19 weeks gestation, aged 16 years or older, sufficiently proficient in English to complete the questionnaires and had a singleton pregnancy.

Specially trained recruitment staff provided a detailed explanation of the prospective nature of the study, including information on an optional consent for biospecimens (maternal and/or infant buccal swab, cord blood, placental biopsy) and permission to access hospital record birth information. Following written informed consent, participants provided a buccal swab and were given the option of completing the first questionnaire (Q1) on site or at home.

Table 1 shows the population of pregnant women presenting for their first appointment while our recruitment staff were in attendance (n = 11732). A total of 6944 women were not approached as they were either ineligible, missed in clinic, in another study, had a non-viable pregnancy, or were considered unsuitable to approach, e.g. having had a recent fetal loss. Almost 4,800 women were approached about the study. The recruitment of abstinent women ceased in April 2012, when the target number was achieved; hence 457 women were not invited to participate when they volunteered information about alcohol abstinence. A further 58 women were not invited because they were moving interstate, overseas or were scheduled to deliver in a non-study hospital.

**Table 1:** **AQUA cohort recruitment in antenatal clinics; 25 July 2011 to 31 July 2012**

	Clinic	Clinic 1	Clinic 2	Clinic 3	Clinic 4	Clinic 5	Clinic 6	Clinic 7	Total
Women approached	*Participated and completed Questionnaire 1*	*242*	*190*	*99*	*563*	*472*	*478*	*106*	*2146*
	*Did not participate (reason):*								
	Declined; too busy; not interested	136	91	185	127	235	412	52	1238
	Consented, but never returned any questionnaires	93	82	95	197	174	156	88	889
	Abstinent or no alcohol in lifetime*	65	29	0	62	87	207	7	457
	Moving interstate/overseas; delivering elsewhere	5	0	2	5	5	40	1	58
Women not approached	Over 18 weeks gestation	59	58	28	297	421	2797	133	3793
	Less than 16 years of age	1	0	0	1	0	4	1	7
	Multiple pregnancy	11	0	1	23	47	6	1	89
	Not enough English**	124	75	187	333	253	610	42	1624
	Pregnancy not viable	2	0	1	0	0	7	0	10
	Missed in clinic	65	25	16	110	186	579	140	1121
	Already in other research	13	0	0	147	1	37	2	200
	Midwife decision not to approach	5	8	2	14	8	59	4	100
	**Total**	821	558	616	1879	1889	5392	577	11 732

*recruitment of abstinent women ceased on April 12, 2012, when target number was achieved.

**includes three pregnant women with either a hearing, vision or intellectual impairment.

Of those approached, 1238 (27%) declined to participate. In total, 3035 consented to participate, 2146 of whom completed questionnaire 1 (Q1) (71.0%). Of these, 2034 women gave permission to a buccal swab (94.8%), 82.5% to collection of cord blood and placental biopsies at birth, 85.4% to infant buccal swabs and 91.2% to medical records access.

Table 2 divides the non-participating population into three groups, those who declined, those who consented but did not go on to complete Q1 and a mixture of those not approached or not asked to participate for various reasons as stated above. Participating women were slightly older than all three non-participating groups, and less likely to be in the lowest socioeconomic quartile when compared with non-participants. Women who participated were also slightly more advanced in gestation at their first visit than those who initially consented but never returned Q1.

**Table 2:** **Baseline characteristics of pregnant women at AQUA recruitment sites; 25 July 2011 to 31 July 2012**

Variable	Participants (n = 2,146)	Declined (n = 1,238)	Consented, but never participated (n = 889)	Not eligible, missed, other* (n = 7459)
Maternal age, mean (SD)	31.3 (0.1)	30.5 (0.1)^§^	30.1 (0.2)^§^	30.4 (0.1)^§^
Gestational age at first visit, mean (SD)	14.1 (0.1)	14.2 (0.1)	13.6 (0.1)^§^	N/A
Socioeconomic index ;^£^ lowest quartile	117 5.6%	129^‡^ 11.2%	65 8.0%	704^‡^ 10.1%
Socioeconomic index; second quartile	305 14.5%	179 15.5%	102 12.5%	989 14.1%
Socioeconomic index; third quartile	635 30.3%	337 29.2%	256 31.5%	1889 27.0%
Socioeconomic index; fourth quartile (highest)	1042 49.6%	508 44.1%	391 48.0%	3416 48.8%

*This column comprises all women who were not approached (Table 1), 58 women who delivered elsewhere and 457 women who were not invited to participate after April 12, 2012 because they were either lifetime abstainers or had not consumed any alcohol since becoming pregnant or planning to be pregnant.

^£^Index of Relative Socio-economic Disadvantage: A general socio-economic index summarising and ranking a range of information about the economic and social conditions of people and households within a geographic area. The index was calculated from the 2011 Census of Population and published by the Australian Bureau of Statistics.

^§^p < 0.001 when compared to participants, 2-sample t test.

^‡^p < 0.001 when compared to participants, chi 2.

### Data collection

There are six time points at which data are collected. (Table 3).

**Table 3:** **Summary of participant follow-up and data collection**

Follow up timing	Year	Who is invited?	What is the nature of follow-up?	What is being measured?
12-18 weeks gestation	2011-2012	All participants:	Questionnaire 1; Maternal buccal	Demographics, obstetric history, current pregnancy dates, health, alcohol & other lifestyle, family & relationships Alcohol metabolism
26 weeks gestation	2011-2012	All participants:	Questionnaire 2	Diet, health, alcohol & other lifestyle
35 weeks gestation	2011-2013	All participants:	Questionnaire 3	Obstetric complications health, alcohol & other lifestyle
Birth	2011-2013	All participants:	Access to hospital records; Placenta, cord blood, infant buccal	Perinatal information Infant epigenetics
12 months after birth	2013-2014	All participants:	Questionnaire 4	Birth and coping, breast feeding, child health, child development, health, alcohol & other lifestyle, family & relationships, combining work & family
		Subset of participants:	3D photography of child’s face	Craniofacial morphometrics
24 months after birth	2014-2015	All participants:	Questionnaire 5	Child health, child development, health, alcohol & other lifestyle, family & relationships, combining work & family
		Subset of participants:	Developmental assessment	Detailed child development

#### Pregnancy to birth (this stage is completed)

During pregnancy, participants completed three questionnaires: questionnaire1 (Q1) at 12-18 weeks, questionnaire 2 (Q2) at 26-28 weeks, and questionnaire 3 (Q3) at 35 weeks gestation. A paper version of Q1 was included in a recruitment pack. Participants were able to choose between online and paper follow-up; approximately two thirds of the cohort chose to complete subsequent questionnaires online.

Staff attempted to attend all births that occurred after 37 weeks gestation to collect biospecimens (where consent was provided). An on-call roster system, daily birth suite lists and clinical staff assisted collection. If staff were unavailable for specimen collection at birth, two buccal swabs were posted to the mother with instructions on how to collect cheek cells from her infant within 28 days of birth; almost 80% of infant buccals were collected using this method.

Birth outcome data were obtained by accessing electronic medical records. Selected variables were downloaded by a hospital staff member then forwarded to the study team. Data are available for 1405 participants (97.2% of those consented). Some participants were unable to be matched to hospital records because of inaccurate information on their patient record number or where they gave birth at a different hospital than where they had booked for care.

**Figure 1:**
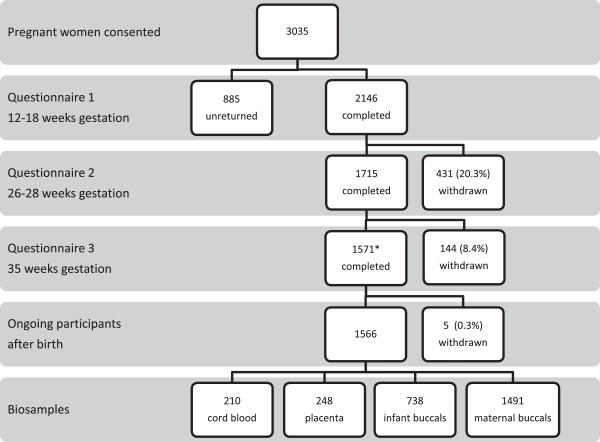
**AQUA cohort participation and attrition from enrolment to birth.** *89.4% (n = 1405) with hospital birth information.

Figure 1 shows the flow of participants through the AQUA study from recruitment to birth. Attrition between Q1 and Q2 was 20.3% with 1715 participants completing both questionnaires. Further attrition of 8.4% occurred between Q2 and Q3 and 1571 participants completed all three pregnancy questionnaires. There was less than 1% attrition between Q3 and birth, resulting in 1566 active participants at completion of pregnancy, 1491 (95.2%) of whom provided a maternal buccal swab. The final biobank contains 248 placental biopsies, 210 cord blood samples and 738 infant buccal swabs. Table 4 details the reasons for attrition at different time points from Q1 until birth. The main reason for attrition was loss to follow-up and a small percentage of women actively withdrew their participation on follow-up (3% of reasons overall). Some women experienced a fetal loss, most early in pregnancy, and 20 participants were withdrawn for administrative reasons, such as lost forms or scheduling errors.

**Table 4:** **AQUA cohort attrition between pregnancy questionnaires**

Reason for attrition	After Q1	After Q2	After Q3	Birth
Dropped-out (loss to follow-up)	383	138		
Withdrew	11	3	3	
Fetal loss	18	2	2	
Administrative reason (e.g. scheduling error)	19	1		
**Total n = 586**	**431**	**144**	**5**	

#### After birth (this stage is in progress)

Questionnaires are completed when the children are 12 months (Q4) and 24 months (Q5) and a subgroup of approximately 75% of the children (up to 150 per exposure group) are sequentially invited for clinical assessments at 12 months (craniofacial) and 24 months (neurodevelopmental).

### Variables

#### Pregnancy alcohol exposure (PAE)

Following extensive development of the PAE assessment (literature review, expert consultations, focus groups, pilot questions) [19] a set of questions, accompanied by a visual guide, was included in all questionnaires to measure PAE. Assessment of PAE was divided into five time points; at 12 weeks prior to conception, from conception but prior to pregnancy recognition, since pregnancy recognition to gestational week 14, from gestational week 15 to week 25, and from gestational week 25 to (at least) week 36 or birth, whichever occurred first. Drinking type, volume, pattern and frequency for each stage of pregnancy was converted to absolute alcohol (AA) and categorised as abstinent (no alcohol consumed); low (≤20gAA/occasion, and <70gAA/week); moderate (21-49gAA/occasion, and ≤ 70gAA/week ); or high (>70gAA/week, including binge with ≥50gAA/occasion) [11]. One standard drink in Australia is equal to 10 g of alcohol. Final exposure groups were defined using a modified algorithm developed in another related study [11] and participants were allocated to one of eight PAE groups (Table 5).

**Table 5:** **Number of Participants by alcohol exposure group**

Prenatal alcohol exposure group	Participants (%)
No alcohol in lifetime	112 (7.1)
Abstinent throughout pregnancy	495 (31.5)
Abstinent in trimester 1, no more than moderate in trimesters 2 and 3	56 (3.6)
Low in trimester 1, abstinent in trimesters 2 and 3	147 (9.4)
Moderate in trimester 1 abstinent in trimesters 2 and 3	146 (9.3)
Low to moderate in trimester 1, no more than moderate in trimesters 2 and 3	254 (16.2)
High in any or all trimesters, incl. binge on special occasions	178 (11.3)
No more than moderate in any or all trimesters, with binge on special occasions pre-aware	99 (6.3)
**Total**	1570* (100)

*Excluding one participant whose PAE was unable to be categorised due to lack of information.

#### Outcome measures

##### a) Questionnaire data

Primary outcome measures are health and physical development at birth and at 12 months and neurodevelopment at 12 and 24 months. Questionnaire 4 at 12 months and Questionnaire 5 at 24 months collect information relating to sensory processing, functional performance and social-emotional-behavioural issues and competencies of the child. The Infant/Toddler Sensory Profile (ITSP) [20] is a 48-item parent-report questionnaire that assesses sensory processing, as well as determining specific sensory profile patterns. Emotional and behavioural problems of the child are assessed using the Brief Infant Toddler Social Emotional Assessment (BITSEA), [21] a reliable and valid screen for emotional and behavioural problems and delays in social competence. The Children with Special Health Care Needs Screener is used to identify if any children have one or more functional limitations or service needs as a result of an on-going health condition [22]. This information on child health is complemented by study-specific questions on number and type of hospitalisations, birth defects and one general question on overall child health.

##### b) Clinical assessments

Forming the clinical review group are participants reporting a range of PAE, plus a maximum of 150 sequentially selected controls (those who were abstinent throughout pregnancy), who agree to clinical assessments at 12 and/or 24 months of age.

At 12 months, three dimensional (3D) facial images of the children are captured [23]. Previous research found a difference in minor facial anomalies between infants born to women who abstained from alcohol and women with low alcohol intake [24]. We are using 3D imaging to analyse detailed facial morphometrics amongst the major PAE groups to identify subtle manifestations of PAE not evident clinically.

At 24 months of age, the clinical review group is offered a neurodevelopmental assessment of their child. This involves administration of the Bayley Scales of Infant and Toddler Development, Third Edition (Bayley-III), [25] which is the most widely used measure of developmental delay in clinical and research settings. The Bayley-III provides an objective assessment of cognitive, language (expressive and receptive), and motor (fine and gross) development, and will be administered by psychologists in the hospital setting who are trained to use this scale.

#### Contextual factors

**Figure 2:**
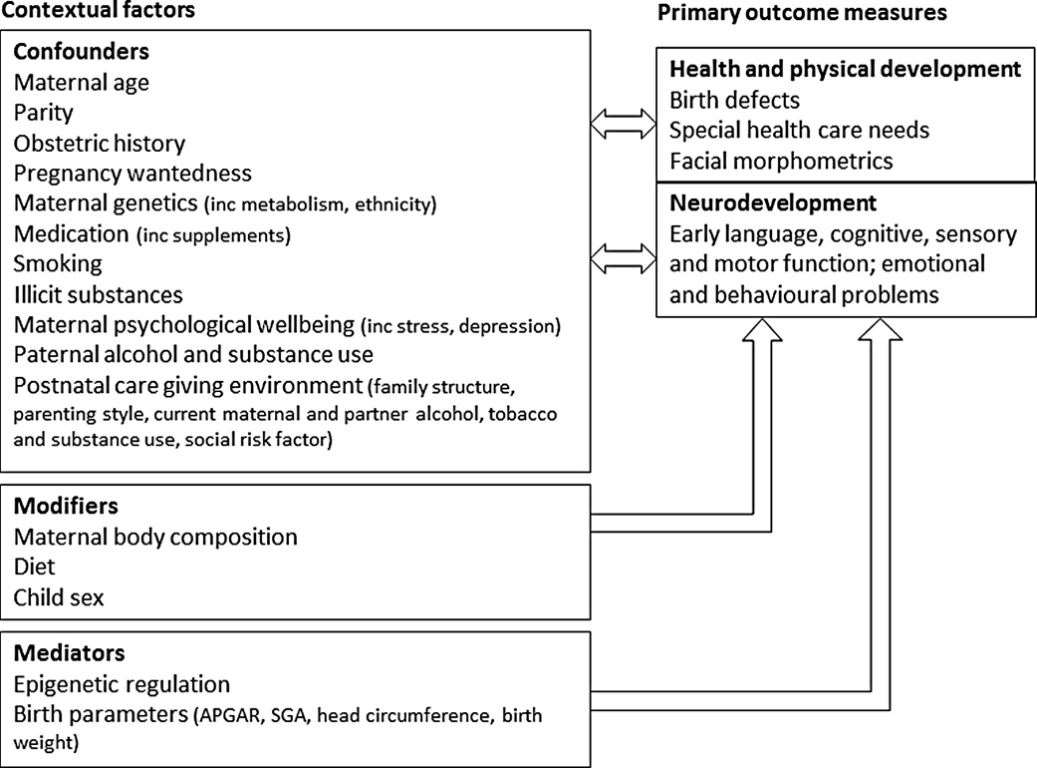
**Contexual factors and primary outcome measures of the AQUA study.**

Careful consideration was given to inclusion of relevant contextual factors previously reported in the literature to influence these outcomes, either as confounders, effect modifiers or mediators (Figure 2). Specific instruments and other measures previously reported in the literature were used where possible, to assess contextual factors as well as outcomes (Table 6).

**Table 6:** **Overview of instruments and measures used in the AQUA study (under copyright and/or published)**

Items	Questionnaire 1	Questionnaire 2	Questionnaire 3	Questionnaire 4	Questionnaire 5
**Family history of alcohol problems**	Family Tree questionnaire (FTQ)(modified) [60]				
**Maternal psychological well being**	Assessment of Quality of Life (AQoL-6D) [61]	AQoL-6D	AQoL-6D	AQoL-6D; Depression Anxiety Stress Scales (DASS, 21 items); [62] Mother-to-Infant Bonding Scale [63]	DASS
**Diet**		Dietary Questionnaire for Epidemiological Studies (DQES v2)^†^			
**Family structure & relationships**				McMaster Family Assessment Device (General Functioning Subscale; [64] LSAC Reciprocal Support for Parenting, Argumentative relationship; [65] List of Threatening Experiences (modified); [66] Quality of Co-parental Interaction Scale (Conflict Subscale) [65, 67]	As in Questionnaire 4
**Parenting style**				Global rating of self-efficacy; [65] Child Rearing Questionnaire (6/30 items); [65, 68] Hostile Parenting Scale [69]	As in Questionnaire 4
**Social disadvantage**				Social risk index; [52] LSAC Social Support and Community Connectedness; [65]	As in Questionnaire 4
**Child health**				Children with Special Health Care Needs Screener (CSHCN) [22]	As in Questionnaire 4
**Neurodevelopment**				Brief Infant Toddler Social Emotional Assessment (BITSEA);^††^ Infant/Toddler Sensory Profile (ITSP)^†††^	As in Questionnaire 4

^†^The authors thank Professor Graham Giles of the Cancer Epidemiology Centre of The Cancer Council Victoria, for permission to use the Dietary Questionnaire for Epidemiological Studies (Version 2), Melbourne: The Cancer Council Victoria, 1996.

^††^Infant-Toddler Social and Emotional Assessment (BITSEA). Copyright © 2000 Yale University and University of Massachusetts. Computer adaptation Copyright © 2012 Yale University and University of Massachusetts. Reproduced with permission of the publisher NCS Pearson, Inc. All rights reserved.

^†††^Infant/Toddler Sensory Profile. Copyright © 2002 NCS Pearson, Inc. Computer adaptation Copyright © 2012 NCS Pearson, Inc. Adapted and reproduced with permission. All rights reserved.

##### a) Confounders

Factors reported in the literature to be both predictors of PAE and adverse child outcomes associated with PAE, but not on the causal pathway are: maternal age 30 years and older; [26] pregnancy wantedness; [27] increasing parity [28] and obstetric history [3]. Further potential confounders collected as repeated measures across all questionnaires include maternal psychological wellbeing, [26–30] smoking, [28, 31, 32] illicit substances, [27, 33] medication and supplement use, particularly folate [14, 34]. Several factors are measured in Q4 (at 12 months after birth), providing proxy measures for unknown antecedent confounding factors. These are maternal depression, the postnatal care-giving environment (based on family structure and parenting style) [26, 31], current maternal and partner tobacco and substance use [26] and social disadvantage [35].

Metabolic processes that modify blood alcohol levels have been shown to be highly variable among individuals, pointing to underlying genetic and environmental differences [36]. Genetic factors, specifically polymorphisms in the alcohol dehydrogenase (ADH) gene family, increase alcohol elimination rates thereby reducing fetal alcohol toxicity by lowering the maternal blood level more quickly than usual [37]. Variants in alcohol metabolising genes also influence a person’s level of alcohol consumption and risk for alcoholism [38]. Further, family history data and ethnic differences in alcohol sensitivity point towards a substantial role for genetic polymorphisms in modifying the adverse effects of maternal alcohol use on pregnancy outcomes [39]. Therefore, maternal and paternal ethnicity were collected in Q1 and genetic polymorphisms (eg *ADH1B*) [40] are assessed in maternal buccal DNA.

##### b) Effect modifiers

We will also consider effect modifiers not directly associated with prenatal alcohol use, but potentially altering the effect of PAE. These are maternal body composition and diet and the child’s sex [28, 41]. Self-reported measures of pre-pregnancy body composition were collected in Q1. A food frequency questionnaire was completed to assess dietary micro- and macronutrient intake as part of Q2 and details of the child’s sex was obtained via hospital birth record linkage.

##### c) Mediators

Epigenetic regulation may be an important mediator on the causal pathway between alcohol exposure and child outcomes [42, 43], reviewed in [44]. Animal studies have shown that PAE leads to changes in global DNA methylation levels and methylation at specific genes, in particular imprinted genes, which are understood to be particularly sensitive to environmental factors [45–48]. DNA methylation will be measured in DNA from infant buccal cells, cord blood mononuclear cells and placental biopsies at gene-specific, global and genome-wide levels.

Further, adverse perinatal outcomes such as preterm birth, small head circumference, low Apgar scores and low birth weight have been associated with high dose alcohol consumption [3, 32, 49] and could be intermediate variables on the causal pathway between PAE and neurodevelopmental problems. Perinatal information was obtained from hospital birth record linkage and in Q4.

### Sample size considerations

#### Facial morphometrics

150 participants in six exposure groups will have 80% power to detect a difference of 0.33 standard deviations between mean values in the abstinent group compared with the exposed groups (effect size of 0.33). Our measures are based on those shown to distinguish between partial FAS and controls [50]. In a related study, [51] the effect sizes for these measures were estimated for FAS versus controls: minimal frontal breadth 0.7, bitragal breadth 0.6, midfacial depth 0.6, total facial height 0.4. Effect sizes for partial FAS (low to moderate doses) are expected to be smaller.

#### Neurodevelopmental assessments

At 12 months of age, there will be high power to detect clinically significant differences in parent report measures of child social, emotional and sensory development, with 150 available in each PAE group. At 24 months of age, with 150 children in each group being clinically reviewed using the Bayley III, there will be 80% power to detect a difference of 0.33 between groups. As the Bayley has a mean of 100 and SD 15, this effect size equates to a difference of 5 points on the scale, a clinically important difference.

### Statistical methods

The effect of different levels of PAE on facial morphometrics and neurodevelopmental assessment scores will be investigated using multivariable linear regressions with alcohol exposure group as a categorical predictor variable. A selection of the possible confounders will be included as independent variables in the regression models - in this analysis of low and moderate PAE, even distribution of many of the confounders across PAE groups can be expected. Adjustments will only be made where groups are unbalanced. In addition, it is likely that there will be a high degree of correlation between many of the possible confounders. If necessary, we will reduce the list of confounders to a smaller group of relatively independent variables through investigation of the correlation patterns, and through grouping techniques such as with the Social Risk Index [52].

### Ethical approval

The establishment of the AQUA study was approved by the Eastern Health Research and Ethics Committee (E54/1011) and the Human Research Ethics Committees of Mercy Health (R11/14), Monash Health (11071B), the Royal Women’s Hospital (11/20) and the Royal Children’s Hospital (31055A). The latter also approved all follow-up of the children (31055C/D).

## Discussion

This longitudinal cohort study seeks to provide new evidence on the complex effects of low to moderate alcohol consumption in pregnancy on early child development by utilising a specifically developed and tested set of questions on alcohol intake. Findings are expected to show how far key mediators and confounders may contribute to the association between PAE and child health and development. Contradictory findings of other studies may be attributed to different or inadequate measures of timing, pattern of use and dose of alcohol consumption. To assess a potential effect of even the lowest levels of prenatal alcohol consumption, we have chosen two reliable early outcomes (facial morphometrics and Bayley-III) which are also feasible and cost-effective in a research setting spanning four years.

As part of our collaboration with expert scientists, clinicians and policy makers at State and Federal Government level, it is planned that we will seek consent for record linkage to school entrance health and development assessments. Further funding will also be sought for detailed investigations into the role of epigenetics, an important mediator in the effect of prenatal alcohol on child outcomes.

Results of this study are expected to substantially contribute to policy review and development, health professional education and consumer information about alcohol consumption for pregnant women in the future. Information will be particularly relevant to anxious women who have consumed alcohol before knowing they were pregnant.

### Strengths and weaknesses

The key strength of the AQUA study lies in its prospective exposure assessment through use of specifically developed and tested questions on alcohol intake that assess timing, pattern of use and dose of alcohol consumption. Our facial morphometric and neurodevelopmental measures are suitable for use in a clinical setting, thereby having the potential to assist in the preclinical diagnosis of FASD, allowing for early treatment to minimise adverse secondary outcomes in later life. We have chosen detailed assessments of emotion and behaviour, as well as cognition, language and motor functioning, these being typically affected in children with FASD. Another novel aspect of this study is the examination of sensory functioning. Development is largely dependent on sensory experiences, and challenges in the processing and integration of sensory information may be a marker for later neurodevelopmental problems. Overall, the combination of questionnaire and hospital record data, clinical assessments and an ability to make correlations with genetic and epigenetic data from stored biospecimens place the study in a unique position to investigate the effect of low to moderate PAE. We are also able to specify PAE prior to pregnancy recognition, a critical phase of embryonic development and before most women cease or reduce their alcohol consumption.

A limitation of any study measuring PAE is that there are currently no validated objective measures to detect low to moderate exposure. Ethanol metabolites in maternal urine, hair or umbilical blood and meconium samples have been used to identify moderate to heavy drinkers around the time of birth with reasonable certainty; [53–55] however, absence of these biomarkers does not provide definite evidence of abstinence. Therefore, we must depend on accurate maternal recall and reporting. However, our focus group work (paper in preparation) indicated that women would answer as accurately as possible, due to their vested interest in the outcomes of this study examining what may be considered normal, non-risky drinking habits.

Further, the validity of some covariates (e.g. body mass, diet, smoking and other lifestyle factors including paternal exposures) may be subject to reporting bias due to a desire to provide socially acceptable responses [56, 57].

While certain facial phenotypes are known to be associated with prenatal alcohol exposure, it is likely that age and ethnicity play a role in our assessment of facial features. To remove a potential age effect, 3D images were captured within two weeks of the child’s birthday. However, it may be difficult to determine whether some features can directly be attributed to PAE if our population is ethnically diverse [51, 58].

A proportion of children identified with early developmental delay will catch up to peers over time, while some children who are developing age appropriately in early childhood, will encounter problems for the first time later in childhood with increasing demand for higher-order cognitive skills. It is known that the Bayley Scales are moderately predictive of later outcomes, [59] which is to be expected given the inter-individual variability in developmental trajectories.

Finally, in instances where there is no clinical review of the child, we depend on maternal report using validated scales to determine child developmental progress. Although we are using widely accepted and validated scales, maternal subjective assessments introduce informant bias.
